# Emergency Physician Perceptions and Experiences in Acute Pain Management: A Qualitative Interview Study

**DOI:** 10.1016/j.acepjo.2026.100485

**Published:** 2026-08-13

**Authors:** Scott J. Keating, Ann M. Menzie, KD Jacobs, Lauren A. Crowder, Kelly L. Johnston, Scott G. Weiner

**Affiliations:** 1 Vertex Pharmaceuticals, Incorporated, Boston, Massachusetts, USA; 2 IQVIA, Providence, Rhode Island, USA; 3 Department of Emergency Medicine, Brigham and Women’s Hospital, Boston, Massachusetts, USA

**Keywords:** acute pain, emergency department, emergency physician perspectives, pain management, qualitative research, opioid analgesics, non-opioid analgesics

## Abstract

**Objectives:**

Acute pain is the leading cause of emergency department (ED) visits in the United States, yet many patients continue to report pain at discharge despite treatment with analgesics. Although prior qualitative studies have examined patient experiences of pain management following a visit to the ED, less is known about the emergency physician (EP) perspective. This study explored EPs’ perceptions and experiences managing acute pain, including perceived barriers to treatment and the consequences of inadequately managed acute pain.

**Methods:**

Semistructured interviews were conducted to elicit EPs’ perceptions of challenges and barriers to acute pain management in the ED and the patient impacts of inadequately managed pain.

**Results:**

Fifteen EPs practicing in the US participated in interviews. EPs reported tailoring acute pain management strategies based on patient comorbidities, the underlying medical conditions, pain severity, and risk of pain medication-related adverse events, and risk of opioid addiction or dependence. Opioids were viewed as effective, but participants described concerns with prescribing them to at-risk patients, opioid-related adverse events, and associated administrative burdens. Nonsteroidal anti-inflammatory drugs (NSAIDs) and acetaminophen were perceived as having tolerability issues and limited analgesic benefit. Nearly all EPs indicated inadequate pain management can have broad negative effects on patients.

**Conclusion:**

Findings suggest an unmet need for acute pain management medications that provide effective analgesia while minimizing safety concerns, administrative burden, and addiction potential.

**The Bottom Line**

Little is known about how emergency physicians (EPs) view the challenges of managing acute pain in the emergency department (ED). In interviews, 15 EPs described tradeoffs with common pain treatments, such as opioids, nonsteroidal anti-inflammatory drugs, and acetaminophen, particularly the challenge of balancing pain relief with safety concerns. Participants also noted some patients continue to have poorly controlled pain, which can interfere with daily functioning and returning to work. These findings highlight limitations in pain management approaches available at the time of the interviews and the need for additional acute pain treatment options.

## 1 Introduction

### 1.1 Background

There are approximately 150 million emergency department (ED) visits in the United States (US) annually.^1^ Pain is the most common reason for seeking care in the emergency setting and accounts for nearly three-quarters of chief complaints.^2^, ^3^, ^4^ Among patients presenting to the ED with pain-related conditions, the level of reported pain is often significant, with an estimated 70-75% of patients describing moderate-to-severe pain.^5^ Pain relief may remain inadequate even when analgesics are administered in the ED. In a large multicenter study, 75% of patients presenting to the ED with moderate-to-severe pain continued to experience moderate-to-severe pain at discharge despite treatment.^6^ Inadequately managed acute pain is also associated with increased health care resource utilization and costs,^7^ including repeat ED visits for the same episode of pain.^8^^,^^9^

### 1.2 Importance

Insufficient relief from acute pain can substantially affect patients’ health and wellbeing, including prolonged recovery, impaired sleep and mood, interference with ambulation and activities of daily living (ADLs), difficulty returning to work or maintaining productivity, and increased risk of developing chronic pain.^10^, ^11^, ^12^, ^13^, ^14^ Poorly controlled acute pain in older adults has also been associated with adverse health outcomes such as delirium.^15^ In the ED, inadequate pain relief has also been associated with longer ED stays, prolonged inpatient stays among admitted patients, and mortality.^16^

Prior qualitative research suggests that patients seeking acute pain relief in ED settings have concerns about opioid addiction, value clear patient-provider communication, and want involvement in treatment decisions.^17^^,^^18^ These studies also describe challenges after discharge, including underuse of prescribed opioids because of dependence concerns and difficulties obtaining follow-up care or medication refills despite continued pain.^17^

### 1.3 Goals of this Investigation

Acute pain management is complex, and emergency physicians (EPs) must balance effective pain relief with patient safety in routine clinical practice.^19^ To manage acute pain, EPs administer or prescribe analgesics with different mechanisms of action, commonly including opioids, nonsteroidal anti-inflammatory drugs (NSAIDs), and acetaminophen. Multimodal analgesia, which combines both opioids and nonopioid treatments, may improve pain control while reducing adverse effects.^20^ However, there is limited understanding of how EPs experience the challenges and barriers associated with acute pain management in the ED. To address this evidence gap, interviews were conducted with a sample of US-based EPs to characterize experiences and unmet needs associated with acute pain management in the ED.

## 2 Methods

### 2.1 Study Design

This cross-sectional qualitative study used concept elicitation (CE) interviews and thematic analysis to understand EPs’ approaches to acute pain management, perceived barriers to treatment, and views on patient impacts of inadequately managed pain. CE interviews provide in-depth firsthand insights into participants' experiences, perceptions, and priorities.^21^^,^^22^

### 2.2 Study Sample

The study aimed to recruit 15 US-based EPs to participate in individual, semistructured interviews. Eligible participants were required to be in good clinical standing, hold an active medical license, be certified by the American Board of Emergency Medicine, and be responsible for acute pain management in the ED. Participants were identified and recruited by a qualified third-party vendor (Global Perspectives), using established physician panels and databases, with mutual anonymity between the sponsor and participants. Although the composition of these recruitment sources is not publicly available, participants were recruited from diverse clinical settings, including teaching and nonteaching institutions, public and private hospitals, and a range of geographic settings. Eligible participants were further required to have ≥3 years of experience practicing in an emergency setting following completion of a medical residency/fellowship program, have an average caseload of ≥50% adult patients, and spend an average of ≥6 shifts a month in a clinical emergency setting (excluding shifts in a stand-alone urgent care facility).

### 2.3 Study Procedures

Data collection occurred in March 2024. EPs were recruited using purposive sampling, a nonrandom sampling method, that allowed identification of participants who met the predefined eligibility criteria.^23^ Each 60-minute interview followed a semistructured interview guide with open-ended questions about EPs’ clinical backgrounds and ED experience, perceived challenges in acute pain management, and perspectives on the patient impacts of inadequately managed pain.

The interview guide was developed with clinical expert input, including an EP, and reviewed by trained qualitative researchers for clarity and alignment with study objectives. Although no formal validation was performed, the development process together with clinical expert input and review of the interview guide supported face validity before use. Interviews were conducted one-on-one using online videoconferencing software. Audio-recordings were transcribed verbatim and coded using both deductive and inductive approaches to address preidentified concepts and capture new concepts that emerged from the data.^24^ Reporting of study findings adheres to Standards for Reporting Qualitative Research guidelines. Institutional Review Board approval was provided by the WCG Institutional Review Board (study number: 1365464), and study procedures were conducted in accordance with the Helsinki Declaration of the World Medical Association. All interview participants provided verbal consent, and data collection was anonymized to the greatest extent possible.

### 2.4 Analyses

Interview data were evaluated thematically to identify patterns in EP perspectives on acute pain management and the perceived patient impacts of inadequate pain control. All data were coded and analyzed in NVivo (V14) using a consensus-building process in which several team members independently coded the same 3 initial transcripts and compared coding to promote coding consistency. Discrepancies were discussed until consensus was reached.

### 2.5 Concept Saturation

Following coding and analysis, the study team assessed concept saturation, defined as the point at which no new concepts emerged from the data.^21^^,^^25^ Transcripts were ordered chronologically and grouped into 5 sets of 3 interviews, and the first appearance of each concept was assigned to the appropriate set. Nearly all concepts (96%) were identified by the second set of interviews, and all concepts were identified by the fourth set, indicating that concept saturation was reached, and the sample size (n = 15) was sufficient for the study objectives. Therefore, no further participants were recruited.

## 3 Results

### 3.1 EP Demographic and Experience Characteristics

A total of 15 EPs participated in qualitative interviews; more than half were male (n = 9, 60.0%) and around half (n = 8, 53.3%) identified as White (Table 1). Study participants predominantly practiced in the Northeast (n = 11, 73.3%), had a median of 23.0 years of experience working in an emergency setting postresidency/fellowship (range from 11.0 to 29.0 years), and worked a median of 14.0 shifts per month in a clinical emergency setting and/or performing clinical tasks (range 8.0 to 20.0 shifts).

**Table 1:** Emergency physician demographics and clinical practice characteristics.

Demographic characteristics	Emergency physicians (N = 15) n (%)
Sex	
Male	9 (60.0)
Female	6 (40.0)
Race/Ethnicity	
White	8 (53.3)
Asian American or Pacific Islander	4 (26.7)
Black or African American	1 (6.7)
Hispanic or Latino	1 (6.7)
Prefer not to answer	1 (6.7)
Geographic region	
Northeast (CT, MA, NJ, NY, RI)	11 (73.3)
South (FL, TX, GA)	3 (20.0)
West (CA)	1 (6.7)

**Clinical experience & characteristics**	**Median (range)**
Years of experience postresidency/fellowship	23.0 (11-29)
Number of clinical shifts per month	14.0 (8-20)
Percentage of adult patients	90.0 (70-98)

CA, California; CT, Connecticut; FL, Florida; GA, Georgia; MA, Massachusetts; NJ, New Jersey; NY, New York; RI, Rhode Island; TX ,Texas.

Note: Listed US states represent emergency physician-reported location of current clinical practice.

All EPs reported working shifts within a hospital-based ED (n = 15, 100%); among this group, 8 (53.3%) provided care at community medical centers, 6 (40.0%) provided care in academic medical centers, and 1 participant did not report the hospital type.

EPs estimated that a median of 60.0% of patients (range 25.0% to 99.0%) presented to the ED with acutely painful conditions or complaints. Almost three-quarters reported that at least half of these patients described their pain as severe. All EPs reported evaluating patients presenting with traumatic injuries (eg, injuries resulting from a fall or motor vehicle accident), including bone fractures, ligament strains, and lacerations. Although asked about their overall approaches to acute pain management, EPs also reported treating a range of other acute pain conditions. These included abdominal, gastrointestinal, urological, gynecological, and cerebrovascular conditions (eg, stroke), headaches and migraines, general chest pain, acute back pain or muscle strains, and sickle cell crises.

### 3.2 EPs’ Role and Approach in Acute Pain Management

All EPs reported being primarily responsible for patients’ pain management in the ED and for prescribing pain medications at discharge. Thirteen EPs reported coordinating acute pain management with other health care providers, most commonly nurses. Around half coordinated with advanced practice providers (APPs); however, some noted that APPs functioned independently in the ED, with EPs being consulted as needed.

When managing acute pain, 12 EPs described focusing on reducing their patient’s pain to a tolerable level, as fully eliminating pain was not considered realistic. Nine EPs reported following self-developed pain protocols, which were informed by their medical training and personal experiences; however, they noted these protocols were informal and varied based on the type of condition being treated, patient characteristics, and familiarity with certain medications. Around half noted their facility had protocols in place for pain management, though they were often condition-specific, including clinical pathway protocols (eg, sickle cell pathway protocol), algorithm-driven recommendations, monitoring requirements, and formulary limitations.

### 3.3 Areas of Focus for Pain Management in the ED

When planning pain management regimens, EPs described key considerations, including past and current medical conditions, pain severity, risk of adverse effects (AEs), and patient preferences. Nearly all EPs reported considering comorbidities, including kidney failure or metabolic disorders, mental health conditions, obesity, and hypotension (Table 2). EPs also reported considering pain severity and the underlying condition when selecting treatment, including conditions such as sickle cell crisis, bone fracture, kidney or gallbladder stones, back pain or muscle strain, and dental pain.

**Table 2:** Patient-related characteristics considered by emergency physicians in acute pain management.

	n (%)
Comorbidities	14 (93.3)
Type of medical condition	14 (93.3)
Pain severity	14 (93.3)
Medication interactions, allergies, or tolerability	12 (80.0)
Potential medication-related AEs	10 (66.7)
Opioid misuse	9 (60.0)
Age	8 (53.3)
Considerations of medication administration route	7 (46.7)
Patient-reported satisfaction with care	4 (26.7)
Chronic pain and opioid experience	4 (26.7)
Other substance use	2 (13.3)

AEs, adverse events.

Two-thirds (n = 10) of EPs described challenges managing acute pain among medically complex patient populations, including those with chronic pain, opioid tolerance, or a history of opioid use disorder. Similarly, one-third of EPs noted issues balancing pain relief with the risk of AEs. Further, approximately half of EPs (n = 7) described incorporating patients’ pain management preferences into their approach, while 5 EPs noted they only consider patients’ medication preferences in certain contexts. For example, patients with a history of opioid use disorder may request specific classes of pain medications to avoid risk of returning to opioid misuse.

### 3.4 Emergency Physician-Perceived Impacts of Inadequate Pain Management

Nearly all EPs (n = 13) discussed their perceptions of the impact of inadequately managed acute pain, with more than half (n = 8) of EPs reporting broad and debilitating AE on patients (Table 3). Further, EPs noted negative impacts on the ability to perform ADLs, return to work or productivity, and social functioning and relationships.

**Table 3:** Emergency physician-perceived patient impacts of inadequate pain management.

	n (%)
Broad adverse effects	8 (53.3)
Ability to perform ADLs	6 (40.0)
Return to work or productivity	5 (33.3)
Social functioning and relationships	4 (26.7)
Short, brief, or limited adverse impact	3 (20.0)
Emotional functioning	2 (13.3)

ADLs, activities of daily living.

### 3.5 Perceived Advantages and Disadvantages of Acute Pain Medications

When asked how satisfied they were overall with the medications available for managing acute pain in adults at the time of the interviews, one-third (n = 5) of participants reported they were satisfied; however, the majority were only somewhat satisfied (n = 9). Satisfaction was based on physician self-report. Similarly, when asked about their perceptions of patient satisfaction with pain medications available, 10 EPs felt their patients were at least somewhat satisfied. Accordingly, EPs provided descriptions of advantages and disadvantages of available acute pain medications (ie, opioid medications, NSAIDs, and acetaminophen), including analgesic effectiveness and potential AE (Table 4). Table 4 summarizes medication advantages and disadvantages as described by participants and does not represent a comprehensive list of pharmacologic properties for each medication class.

**Table 4:** Emergency physician-reported pain medication advantages and disadvantages.

	Opioids	NSAIDS	Acetaminophen
Advantages			
Analgesic effectiveness	X	X	X
Ability for intravenous administration		X	
Nonopioid/nonaddictive		X	X
Safety/tolerability in select patients			X
Disadvantages			
Risk of medication-related AEs	X	X	
Addiction, dependence, or diversion risk	X		
Challenges prescribing to at-risk populations	X	X	
Tolerability or contraindication concerns	X	X	
Modest analgesic effect compared with opioids		X	X
Impacts on liver function			X
Risk of overdose	X		

AEs, adverse events; NSAIDs, nonsteroidal anti-inflammatory drugs.

### 3.6 Opioid-specific Considerations

Six EPs reported being comfortable prescribing opioids, whereas 8 reported varying levels of comfort; one participant was uncomfortable prescribing opioids. An advantage of opioid medications, as described by over half of EPs, was effectiveness in managing acute pain (Table 4). Conversely, participants reported multiple disadvantages of opioid medications, including prescribing challenges in at-risk populations, such as the elderly or those with respiratory comorbidities.

They also described opioid-related adverse drug events (ORADEs), including drowsiness, sedation, altered cognition, constipation, respiratory depression, and the potential for overdose. Nearly all EPs expressed concern about the risk of addiction, dependence, and diversion. Four EPs indicated that prescribing short courses of opioids may help mitigate these risks.

All participants described administrative factors or tasks involved in prescribing opioid medications, such as state monitoring programs and the need to navigate formulary restrictions or quantity limits. Some EPs experienced prescribing limitations due to stock shortages. About half of EPs reported these requirements as burdensome, whereas others felt they were manageable and served an important role in reducing opioid misuse risk.

### 3.7 Nonopioid Pain Medication Considerations

Seven EPs reported NSAIDs were effective in reducing acute pain and inflammation as a first-line treatment (Table 4). Other reported advantages of NSAIDs included intravenous administration and their nonaddictive, nonopioid properties. In contrast, nearly all EPs (n = 14) reported disadvantages of NSAIDs, including risk of AEs (eg, renal failure or gastric bleeding), along with challenges prescribing NSAIDs to at-risk patient populations, such as those with renal conditions or gastrointestinal issues.

EPs also described acetaminophen as a treatment for acute pain. Seven cited at least 1 advantage, including analgesic benefit, nonaddictive properties, and safety or tolerability for select patients. However, 9 EPs identified disadvantages, most notably liver-related concerns and modest analgesic effectiveness (Table 4).

### 3.8 Other Analgesics and Nonpharmaceutical Acute Pain Management Methods

Although nearly all EPs discussed acetaminophen, NSAIDs, and opioids, alternative pharmacologic and nonpharmacologic approaches were mentioned less often. These included anxiolytics or sedatives (n = 6), select adjunctive therapies (eg, topical agents [n = 4], nerve blocks [n = 2], ketamine [n = 5]), and nonpharmacologic strategies, such as heat or ice and limb support.

EPs valued ketamine, sedatives, nerve blocks, and muscle relaxants for selected circumstances but described limitations, including monitoring requirements, AE, short duration of effect, and limited outpatient suitability. Given their infrequent mention, these pain management approaches were not a primary focus of the analysis.

### 3.9 Ideal Pain Management Therapies in the ED

EPs described characteristics of an ideal pain medication, including an improved safety profile, absence of drug interactions, good patient tolerability, and nonaddictive or noneuphoric properties (Table 5). Eight EPs emphasized the importance of convenience in medication administration, such as rapid onset, lower required doses, prolonged analgesic effects, and flexibility in routes of administration. Additionally, 7 highlighted the need for high analgesic efficacy. Other desirable features included a novel mechanism of action, nonopioid formulation, and affordability or insurance accessibility.

**Table 5:** Ideal pain medication characteristics.

Ideal pain medication characteristics	n (%)
Improved safety profile, well tolerated, no drug interactions	13 (86.7)
Nonaddictive or noneuphoric	11 (73.3)
More convenient drug administration	8 (53.3)
High analgesic efficacy	7 (46.7)
Inexpensive, covered by insurance	2 (13.3)
Nonopioid	2 (13.3)
Novel mechanism of action	2 (13.3)

Representative quotes supporting these themes are provided in Table 6.

**Table 6:** Concept-specific exemplary quotes from emergency physician participants.

Topic	Quotes
Emergency physicians’ role and approach in acute pain management	*“My goal is not zero pain, my goal is comfortable enough to be able to be more relaxed and to take them out of acute distress from pain.” (EP111)* “*it depends… what the patient is coming in for. So, if it’s a kidney stone usually I start with anti-inflammatories and…if it’s a muscle strain, usually non-steroidals like ibuprofen and then a muscle relaxer. [If] it’s a fracture, then if their blood pressure can tolerate it, then I will probably give them some opiates. [It] just really depends what that complaint is.*” *(EP108)*
Areas of focus for pain management in the ED	*“Patients with a history of chronic pain is [sic] very, very tough. Patients with a history of substance abuse, psychiatric patients…patients who are demented is huge, elderly patient populations with multiple drug interactions, constipation… with general populace, but especially with the elderly are particularly tough.” (EP103)* *“…Adverse reaction in any way; hypotension, potential for abuse, constipation, nausea, vomiting. I want to give them something that works but I don't wanna invite more problems, headaches, concerns, secondary to the medication... I think adverse reactions are a huge [challenge].” (EP115)*
Emergency physician-perceived impacts of inadequate pain management	*“[Patients are] destroyed by inadequate pain management. Inadequate pain management is probably the reason why we have an opiate problem.” (EP113)* *“[P]eople can’t go about their daily activities if they’re in pain, right? You can’t work, you can’t take care of yourself, you can’t interact with your friends and family. You can’t do things that you enjoy doing when you’re in pain. So, people’s lives and lifestyles and quality of life are very negatively impacted by being in pain.” (EP108)*
Perceived advantages and disadvantages of acute pain medications	*“I think that we as, as a medical community, I think that it would be nice to have other options. And the options that we have… are more limited, because a lot of people, they can’t tolerate the NSAIDs, and they also have sometimes, a not-so-great safety profile long-term—and that they can cause GI bleeds and renal dysfunction. So, it would definitely be nice to have other options—now that opioids are considered to be dependent.” (EP107)* *“I think non-steroidals are really good but they have their own side effects. GI upset and a lotta GI potentially issues, especially if the person’s not eating, and then they’re having kidney issues. It’s just problematic. [S]ometimes in acute pain, non-steroidals are just not enough, at least initially. And then with everything going on with opiates, it’s just very, very difficult to be confident in the fact that you’re writing this prescription, and that you’re not compromising your patient in some way… It’s very complicated, especially in an emergency setting where, 1) you’re trying to adequately treat acute pain, and then, 2) you don’t really know these patients.” (EP108)*
Opioid-specific considerations	*“[P]atients with unstable vitals. If they have an acute medical condition that causes systemic effects and as a result their vital signs are unstable, if they're hypotensive or hypoxic secondary to an acute respiratory issue that's also causing pain, it's difficult to administer drugs that may also make the patient more hypotensive or suppress their respiratory status further. So, that's difficult.” (EP106)* *“…I try to stay away from opiates. I don’t want someone giving ‘em out to their friends or finding out they like them…they could try it once and become addicted and I try to stay away from—young people, giving them a prescription…” (EP110)* *“[T]he [ePrescription] system kind of verifies that you are the prescriber of these opioids— the system pushes a confirmation to your phone that you have to click and say that you are this person…I accept it as part of the process, but I’d rather not spend those whatever it is, few minutes, doing that process.” (EP109)* “*I think they're checks and balances. And, you know, in the beginning, the state monitoring programs were difficult to access[…] and a barrier, but now with the electronic medical records it’s just one click away and not very difficult to do.” (EP104)*
Ideal pain management therapies in the ED	*“[S]omething that’s a strong enough pain medication, but something that’s not habit forming or addictive or harmful.” (EP101)* *“Definitely patients should be pain free because that's initially why they come to the emergency room, to have their pain treated.” (EP114)* *“Ideally, the medication would affect those pain receptors and give patients adequate acute pain control… without causing neurological analgesia.” (EP108)*

## 4 Limitations

Several limitations should be considered when interpreting these findings. First, there is a potential for selection bias, as physicians who chose to participate may have had stronger opinions about acute pain management or greater professional interest in the topic than those who did not participate. Although purposive sampling across multiple recruitment channels was used to support diversity in the sample, more than two-thirds of participants were from the Northeastern US. This geographic concentration may limit transferability to other regions or practice settings. In addition, because purposive rather than probabilistic sampling was used, the findings are not statistically generalizable, although they may be transferable to similar contexts. Second, all findings were based on self-reported data and may therefore be influenced by social desirability bias. To reduce this risk, interview questions were framed neutrally and open-ended, and participants were assured their responses would remain anonymous and confidential.

## 4 Discussion

This qualitative study explored the EPs’ perspectives on acute pain management in the ED, including perceived barriers to treatment and the consequences of inadequately controlled pain on patients. Participants reported a substantial proportion of ED visits involve acutely painful medical conditions, consistent with prior research.^2^, ^3^, ^4^ EPs described treating a broad range of acutely painful conditions, most commonly traumatic injuries, but also conditions with complex pathophysiology or associated complications, such as acute low back pain or sickle cell-related pain. These scenarios may be particularly challenging because clinicians must balance effective analgesia with medication-related risk.^26^^,^^27^

EPs described using both nonopioid analgesics, including NSAIDs and acetaminophen, and opioids to manage moderate-to-severe acute pain in the ED.^28^^,^^29^ Most participants were only somewhat satisfied with pain medications commonly used in routine ED practice during the study period, and described important tradeoffs across treatment options, particularly the challenge of balancing pain relief with safety. These findings suggest that dissatisfaction reflected the limitations and tradeoffs associated with therapies commonly used in ED practice during the study period, rather than a simple lack of treatment options. Participants frequently raised concerns regarding ORADEs, including respiratory depression, constipation, and altered cognition. These perceptions are consistent with prior literature identifying ORADEs as common and clinically important complications of opioid therapy.^30^, ^31^, ^32^ For example, qualitative research conducted outside of the US, EPs described “fear” of adverse events when administering opioids, such as respiratory depression, as a barrier to effective opioid use in emergency care.^33^

Even short-term opioid use has been associated with risk of ORADEs, which may adversely affect patient outcomes and increase health care resource utilization, costs, and mortality.^30^ In the present study, EPs’ concerns about these risks appeared to shape both prescribing decisions and overall satisfaction with acute pain treatment options discussed by participants.

Nearly all participating EPs also expressed concern about opioid addiction, dependence, or diversion. In a recent study of 29 geographically diverse EDs, opioid use among adult trauma patients was associated with a significantly increased risk of “at-risk opioid use” within 3-months, and patients receiving an opioid prescription at ED discharge had a nearly 5-fold risk compared with patients not receiving opioids.^34^ These concerns are also reflected in qualitative research, which suggests that some patients may underuse prescribed opioids or feel apprehension about taking them because of fear of addiction.^17^^,^^18^^,^^35^

These findings should also be interpreted in the context of a rapidly evolving opioid policy environment. Over the last decade, pain treatment guidelines and prescribing practices have changed substantially, including the introduction of state-level limits on the duration and quantity of initial opioid prescriptions.^36^, ^37^, ^38^ Broader policy efforts have also encouraged the use of nonopioid alternatives in ED settings.^39^ Although opioid prescribing practices have historically varied across EDs and individual providers,^40^^,^^41^ the impact of recent utilization management policies on addiction rates remains uncertain.^38^^,^^40^

All participating EPs reported administrative burdens associated with prescribing opioid medications, including state monitoring programs, formulary restrictions, and quantity limits. These requirements result in increased administrative burden that may contribute to provider burnout,^42^ a phenomenon disproportionately affecting EPs,^43^^,^^44^ and may also delay timely pain treatment.^17^ In this context, participants’ desire for treatment options with lower administrative complexity is understandable and clinically relevant.

EPs valued NSAIDs and acetaminophen for their nonaddictive mechanisms of action but reported mixed perceptions of their effectiveness for moderate-to-severe acute pain. Participants also identified important safety concerns, including hepatotoxicity for acetaminophen and renal dysfunction or gastrointestinal bleeding for NSAIDs. These risks are consistent with the published literature,^31^ and underscore the need to balance analgesic effectiveness with medication-related risk through patient-centric treatment strategies.

EPs infrequently described other nonopioid or nonpharmacologic approaches used in multimodal acute pain management, including ketamine, sedatives, nerve blocks, and muscle relaxants. Although these approaches were valued for pain reduction, sedation, or dissociative effects, participants also described limitations, such as short duration of effect, adverse events, and restrictions related to administration or outpatient use. Because the interviews were conducted in March 2024, participant perspectives reflect the acute pain treatment landscape before approval of newer nonopioid therapies, such as suzetrigine, an oral, selective voltage-gated sodium channel 1.8 (Na_V_1.8) pain signal inhibitor approved in the US in January 2025 for the management of moderate-to-severe acute pain in adults.^45^, ^46^, ^47^, ^48^ Future research should evaluate whether therapies, such as suzetrigine, may help address the unmet needs identified by EPs in this study, particularly the need for effective analgesia with fewer safety, administrative, and addiction-related concerns.

Nearly all EPs indicated inadequately managed acute pain can have widespread negative effects on patients, including impaired daily functioning, reduced quality of life, and difficulty returning to work. These findings are consistent with prior literature showing many patients are discharged from the ED while still experiencing significant pain.^5^^,^^6^ Persistent pain after discharge may contribute to ongoing functional impairment and reliance on alternative or suboptimal pain relief strategies.^19^^,^^20^ Together, these findings highlight the importance of timely and effective acute pain management.

The challenges identified by participants contributed to a desire for novel analgesics that are effective, safe, well tolerated, nonaddictive or noneuphoric, and easy to administer. These desired characteristics reflect both the clinical complexity of acute pain management in the ED and the limitations of treatment options available during the study period.

This qualitative study provides important insight into EPs’ experiences managing acute pain in the ED. Participants described barriers to effective pain management, including clinical complexity, medication-related risks, and administrative burden. They also reported that inadequately managed acute pain can substantially affect patients’ functioning and quality of life. Although opioid and nonopioid therapies available during the study period offered important benefits, participants emphasized these options also have meaningful limitations. Overall, findings suggest an unmet need for acute pain treatments that provide effective analgesia while minimizing safety concerns, administrative burden, and addiction potential.

## Author Contributions

SJK and SGW: study concept and design, interpretation of data, drafting/revising the manuscript for important intellectual content. AMM: study concept and design, interpretation of data, drafting/revising the manuscript for important intellectual content, and acquisition of funding. KDJ, LAC, and KJL: study concept and design, acquisition of data, analysis and interpretation of data, and drafting/revising the manuscript for important intellectual content.

## Funding and Support

By *JACEP Open* policy, all authors are required to disclose any and all commercial, financial, and other relationships in any way related to the subject of this article as per ICMJE conflict of interest guidelines (see www.icmje.org). Funding, design, and interpretation of this research, as well as activities surrounding preparation and publication of this manuscript, were provided by Vertex Pharmaceuticals Incorporated.

## Conflict of Interest

Scott J. Keating and Ann M. Menzie are employees of Vertex Pharmaceuticals Incorporated, and may own stock or stock options in the company. Kelly L. Johnston is employed by IQVIA, a consulting firm retained by Vertex Pharmaceuticals Incorporated to conduct research pertaining to this work; KD Jacobs and Lauren A. Crowder are former employees of IQVIA. Scott G. Weiner is an employee of Brigham and Women’s Hospital, which received grants or contracts from the National Institutes of Health, Foundation for Opioid Response Efforts, and Yale University Elevance Foundation; received consulting fees from Vertex Pharmaceuticals Incorporated and Cessation Therapeutics.
